# International Trade Modelling Using Open Flow Networks: A Flow-Distance Based Analysis

**DOI:** 10.1371/journal.pone.0142936

**Published:** 2015-11-16

**Authors:** Bin Shen, Jiang Zhang, Yixiao Li, Qiuhua Zheng, Xingsen Li

**Affiliations:** 1 Ningbo Institute of Technology, Zhejiang University, Ningbo, Zhejiang, China; 2 School of Systems Science, Beijing Normal University, Beijing, China; 3 School of Information, Zhejiang University of Finance and Economics, Hangzhou, China; 4 School of Computer Science and Technology, Hangzhou Dianzi University, Hangzhou, China; East China University of Science and Technology, CHINA

## Abstract

This paper models and analyzes international trade flows using open flow networks (OFNs) with the approaches of flow distances, which provide a novel perspective and effective tools for the study of international trade. We discuss the establishment of OFNs of international trade from two coupled viewpoints: the viewpoint of trading commodity flow and that of money flow. Based on the novel model with flow distance approaches, meaningful insights are gained. First, by introducing the concepts of trade trophic levels and niches, countries’ roles and positions in the global supply chains (or value-added chains) can be evaluated quantitatively. We find that the distributions of trading “trophic levels” have the similar clustering pattern for different types of commodities, and summarize some regularities between money flow and commodity flow viewpoints. Second, we find that active and competitive countries trade a wide spectrum of products, while inactive and underdeveloped countries trade a limited variety of products. Besides, some abnormal countries import many types of goods, which the vast majority of countries do not need to import. Third, harmonic node centrality is proposed and we find the phenomenon of centrality stratification. All the results illustrate the usefulness of the model of OFNs with its network approaches for investigating international trade flows.

## Introduction

With the rapid pace of globalization process, the world economy is increasingly tightly interconnected by global trade flows. International trade in goods has grown from $6.4 trillion in 2002 to $17.8 trillion in 2012 (which is 24 percent of the global GDP), and the share is continuing to grow [1]. Countries benefit from specialization and engaging in international trade flows [2]. Evidence [3, 4] has shown that global flows have profoundly impacted on the competitive landscape in various industries and greatly boosted economic growth of various countries. Recent literature [1, 5, 6] has suggested that economic growth of a country can be explained by investigating its position and role in the global trade flow network. Therefore, modeling and analyzing international trade flow network is of primary interest for understanding industrial competitiveness and economic growth of a country, as well as its policy in the world trade system.

Up to now, some studies [1, 7–11] have been carried out towards trade flows. A branch of them focus on explaining bilateral trade flows (i.e., trading partners and trading volumes). For example, Tinbergen [7] and Helpman et al. [10] estimated international trade flows using gravity equations and a bias-corrected gravity model respectively. Manyika et al. [1] examined a much broader scope of flows (which are total global flows of goods, services, finance, people, and data and communication flows), and presented a connectedness index which ranks countries on these flows. Some work tries to analyze trade flows from the viewpoint of the global trade system. For instance, Serrano et al. [11] developed a procedure which is able to extract dominant flows from the international trade web. Although the above studies analyzed trade flows to some extent, a formal model with effective methods is urgently needed for modeling and analyzing trade flow from the viewpoint of networking.

Currently, several types of trade networks have been built and several methods have been introduced for investigating international trade. For example, Hausmann and Hidalgo [5] adopted a bipartite network to connect countries and their export products, and then developed a model for understanding the relationship among country, capability and product. Serrano and Boguñá [12] established the binary and undirected world trade web and presented its structural characteristics, e.g., scale-free inhomogeneities. Fagiolo et al. [13] studied the topological properties and their evolution over time based on a weighted world trade network. Fan et al. [6] explored countries’ roles and positions in international trade using a complex network perspective, and introduced the concepts and methods (such as community structure, importance of vertices and bootstrap percolation), which is commonly used in complex networks, into the study of international trade. However, these efforts do not consider that countries’ bilateral trade flows are intrinsically highly correlated, which typically form international supply chains or value-added chains.

Therefore, in this paper, we introduce open flow networks and flow-distance based methods [14] into the study of international trade, which offer a new perspective and effective tools for investigating countries’ roles and positions in the global supply chains. Open flow network (OFN) is a useful tool for describing and analyzing open flow systems in a wide spectrum of application domains, such as ecology [15–17], traffic [18] and transportation [19]. OFNs are commonly modelled by directed weighted networks, where directions and weights of edges represent directions and volume fluxes of flows respectively. Because open flow systems always exchange energy, matter and information with their surroundings, flow networks normally have two special nodes (i.e., the source and the sink) representing the environment, where all flows are supposed to run from the source, through the flow system and finally to the sink node [14].

Based on the model of OFNs, many useful techniques have been developed for exploring structures and dynamics of flow systems. For example, Ahuja et al. [20] summarized the classical concepts, algorithms and applications pertaining to flow networks, such as shortest path finding and network optimization. Rosvall and Bergstrom [21] proposed a method of probability flow of random walks for revealing community structure in weighted and directed networks. Vézina and Platt [22] described an inverse method for estimating network fluxes in undersampled environments. Ser-Giacomi et al. [19] defined network entropies, which are related to the statistics of stretching in the fluid, and used a community detection algorithm to partition the network into coherent regions. In a recent work, Guo et al. [14] solved a fundamental problem of how to measure the distances between nodes in flow systems, and put forward several flow distances, such as the first-passage flow distance and the total flow distance. In this study, we introduce these effective approaches developed for OFNs (such as flow distances) into the domain of international trade, and explore new features of open trade flow networks.

Overall, from the new perspective of OFNs, we establish the model of open flow networks of international trade. The progress of calculating flow distances between nodes enables us to understand countries’ roles and positions in the international supply chains and value-added chains. Meaningful insights are obtained from OFNs of international trade and are reported in this study. This work illustrates the usefulness of the model of OFNs with its network approaches for the study of international trade, and provides new insights into global trade flows.

The rest of the paper is structured as follows. Firstly, a formal description of open flow networks is presented, and various types of flow distances are introduced. Then, we discuss the establishment of OFNs of international trade from two coupled viewpoints: the viewpoint of trading commodity flow and that of money flow. Thus, using the approach of flow distances, some obtained results on countries’ trophic levels, niches and centralities are discussed. Here, trading trophic levels and niches are used to quantitatively measure countries’ roles and positions in the international supply chains (or value-added chains). Harmonic centrality is defined to indicate countries’ centralities in the OFNs of international trade. At last, we give the conclusions of this study.

## Materials and Methods

### Model of open flow networks

OFNs are a special type of complex networks, which is able to well depict flows in networks. The definition is described as below.

**Fig 1:**
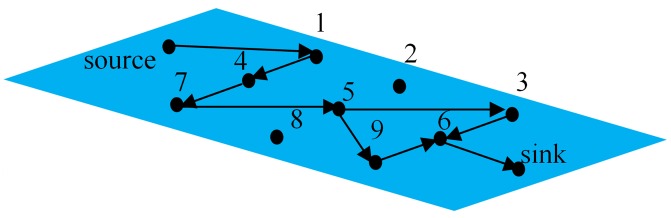
Illustration of an open flow network.

An open flow network is a directed (binary or weighted) graph *OFN* = {*V*, *E*}, where *V* and *E* are the node set and edge set respectively. The node set *V* is supposed to contain *N* common nodes and two special nodes “source” and “sink”, where “source” is the start of all flows and denoted as node 0, and “sink” is the end of all flows and labeled as node *N* + 1. The edge set *E* can be written as an adjacency matrix as below.

$$\begin{array}{c} E={\left\{e_{i,j}\right\}}_{\left(N+2)\times (N+2\right)};\;\;i,j\in \left\{0,\cdots ,N+1\right\}. \end{array}$$ (1)

where *e*_*i*,*j*_ is the flow from node *i* to node *j*. Especially, the first column and the last row of this matrix are all 0 because there are no inflow to “source” node and no outflow from “sink” node. Normally *e*_0,*N*+1_ is 0, because ordinarily there is no flow from “source” to “sink” directly. The total inflow to node *j* (denoted as *e*_⋅*j*_) is calculated as $\sum_{i=0}^{N+1}e_{ij}$ and the total outflow from *i* (labeled as *e*_*i*⋅_) is $\sum_{j=0}^{N+1}e_{ij}$. Because the flow system is assumed to be in equilibrium, except “source” and “sink”, all other nodes have a balanced inflow and outflow, that is *e*_⋅*i*_ = *e*_*i*⋅_, where *i* = 1, ⋯, *N*. The flows to “sink” (i.e., *e*_*i*,*N*+1_) are regarded as *dissipation*. An illustration of OFN is given in Fig 1.

### Distances on open flow networks

In [14], L. Guo et al. presented flow distances on open flow networks, which are given as below. Besides, a symmetric minimum flow distance is also proposed in this subsection.

Given an *OFN*, suppose a large enough number (say *λ*) of particles flow along directed edges randomly. This random walk process can be assumed as a Markov chain. *The mean first-passage flow distance* (MFPFD) from one node *i* to another node *j* (denoted as *l*_*ij*_) is defined as the expected number of steps for reaching *j* for the first time, given that initially the particles are at *i*. *The mean total flow distance* (MTFD) (denoted as *t*_*ij*_) is the average number of steps for arriving at *j* regardless of whether it is the first time of arriving, also given that the particles are at *i* initially.

To illustrate the above concepts vividly, the following experiment is designed and considered. Suppose all particles are initially not colored. There are two situations. 1) If particles go through node *i*, their color turns blue; then when blue particles arrive at node *j*, they become red. In this situation, MFPFD from *i* to *j* is the average number of steps of blue particles turning red. 2) If particles go through node *i*, their color turns blue; blue particles remain blue, when arriving at node *j*. Then, MTFD from *i* to *j* is the average number of steps of all accumulated blue particles (also containing those whose arrival times is more than 1) arriving *j*. Here, a blue particle may be counted repeatedly if it arrives at *j* for multiple times.

Based on the above experimental description, the computation of flow distances are given as below. Formally, we have a stochastic matrix describing the transitions of the Markov chain $M_{\alpha }={\left\{m_{ij}^{\alpha }\right\}}_{\left(N+2)(N+2\right)}$, where

$$\begin{array}{c} m_{ij}^{\alpha }=\{\begin{array}{cc} \frac{e_{ij}^{\alpha }}{\sum_{j=0}^{N+1}e_{ij}^{\alpha }} & \sum_{j=0}^{N+1}e_{ij}^{\alpha }\neq 0\\ 0 & \sum_{j=0}^{N+1}e_{ij}^{\alpha }=0. \end{array} \end{array}$$ (2)

Then, for this absorbing Markov chain, its fundamental matrix is defined as below:

$$\begin{array}{c} U_{\alpha }={\left(I-M_{\alpha }\right)}^{-1}=I+M_{\alpha }+M_{\alpha }^{2}+\cdots , \end{array}$$ (3)

where *I* is the corresponding identity matrix with the same size of *M*_*α*_. Thus, we have the matrix of MTFD $T_{\alpha }={\left\{t_{ij}^{\alpha }\right\}}_{\left(N+2)(N+2\right)}$ and that of MFPFD $L_{\alpha }={\left\{l_{ij}^{\alpha }\right\}}_{\left(N+2)(N+2\right)}$, where

$$\begin{array}{ccc} t_{ij}^{\alpha } & = & \frac{{\left(M_{\alpha }U_{\alpha }^{2}\right)}_{ij}}{u_{ij}}, \end{array}$$ (4)

$$\begin{array}{ccc} l_{ij}^{\alpha } & = & t_{ij}^{\alpha }-t_{jj}^{\alpha }. \end{array}$$ (5)

The detailed derivations of the above formulas can be found in [14].

$t_{ij}^{\alpha }$ and $l_{ij}^{\alpha }$ are asymmetric and cannot well satisfy the applications, which need symmetric distance metrics (e.g., clustering and generating minimum spanning tree). Therefore, here we present a symmetric flow distance called *symmetric minimum flow distance* (SMFD) $f_{ij}^{\alpha }$ as below.

$$\begin{array}{c} f_{ij}^{\alpha }=\text{min}\left\{l_{ij}^{\alpha },\;l_{ji}^{\alpha }\right\}. \end{array}$$ (6)

These elements $f_{ij}^{\alpha }$ form the matrix of SMFD $F_{\alpha }={\left\{f_{ij}^{\alpha }\right\}}_{\left(N+2)(N+2\right)}$.

Based on the tools of MTFD, MFPFD and SMFD, we may quantitatively investigate countries’ roles and positions in the trade flow networks, which cannot be reached by traditional economic methods.

### Open flow networks of international trade

We use the NBER-United Nations trade data (http://cid.econ.ucdavis.edu/nberus.html) to explore new features of OFNs of international trade. The dataset covers the details of world trade flow from 1962 to 2000, and SITC4 (4-digit Standard International Trade Classification, Revision 4) standard [23] is used to organize hundreds types of products in the dataset. A fragment of the dataset is shown in Table 1, where “ICode” and “ECode” are the corresponding codes for importers and exporters respectively, and “Value” means the value of commodity whose unit is one thousand US dollars. “DOT” (direction of trade) has two options: 1 (data from the importer) and 2 (data from the exporter). The “Quantity” of commodity is measured by “Unit” whose codes can be “W” (weight of metric tons), “V” (volume of cubic meters) and so on. Readers can refer to [24] for a detailed description of this dataset. In this study, we use the value of commodity as the volume flux of the trade.

**Table 1:** A fragment of the NBER-United Nations trade dataset.

Year	ICode	Importer	ECode	Exporter	SITC4	Unit	DOT	Value	Quantity
2000	457640	Thailand	532760	Germany	6832	W	1	157	9
2000	457640	Thailand	532760	Germany	6842	W	1	11524	2731
…	…	…	…	…	…	…	…	…	…
2000	532080	Denmark	211240	Canada	8211	W	1	163	16

Using the data in year 2000, we build OFNs of international trade for different types of commodities. In the OFNs, there are 190 common nodes and two special nodes (i.e., “source” and “sink”), where each common node represents a country. Given a commodity, two types of OFNs of international trade can be established, which are depicted from two coupled viewpoints: the viewpoint of trading commodity flow and that of money flow. For simplicity, we call them *commodity flow network* and *money flow network* respectively. Since there are 1288 types of commodities, 1288 commodity flow networks and 1288 money flow networks can be established. A detailed explanation is given below.

#### Interconvertibility of Commodity flow network and money flow network

Given a commodity, we can obtain a commodity flow network (say *CFN*) and a money flow network (say *MFN*). The structures of *CFN* and *MFN* are coupled, and have the following properties.

*Property 1*. The topologies of *CFN* and *MFN* are the same.
*Property 2*. In *CFN* and *MFN*, the volume fluxes of the corresponding edges are the same, but the directions are completely reversed.

Proof. Properties 1 and 2 can be easily inferred according to the fact that the trading of commodity is always accompanied by money flow with the same value but the reverse direction.

*Property 3*. The source and the sink in these two networks are interchanged.

Proof. Since the flows in *CFN* and *MFN* are reversed and they have the same topology, it is natural to interchange the source and the sink. Thus, in these two networks, all flows can start from the source and end at the sink.

According to Properties 1, 2 and 3, we can say that *CFN* and *MFN* are *interconvertible*. Given a *CFN*, the corresponding *MFN* can be obtained using the following two steps, and vice versa.

Step 1: Reverse the directions of all edges.
Step 2: Interchange nodes “source” and “sink”.

#### Viewpoint of Trading Commodity Flow

The viewpoint of commodity flow depicts the production, the transportation process and the consumption of commodities. In a commodity flow network, nodes “source” and “sink” are the start and the end of all commodity flows respectively. Therefore, from the viewpoint of commodity flow, there are three cases for a directed edge.

Case 1: If there exists a trade relationship between two countries, a directed edge can be built from the exporter to the importer.
Case 2: An edge from “source” to node *j* means the production of this commodity in the country *j*.
Case 3: An edge from node *j* to “sink” represents the consumption of this commodity in the country *j*.

The volume flux of each edge can be set as the corresponding value of the trade, the production and the consumption respectively.

**Fig 2:**
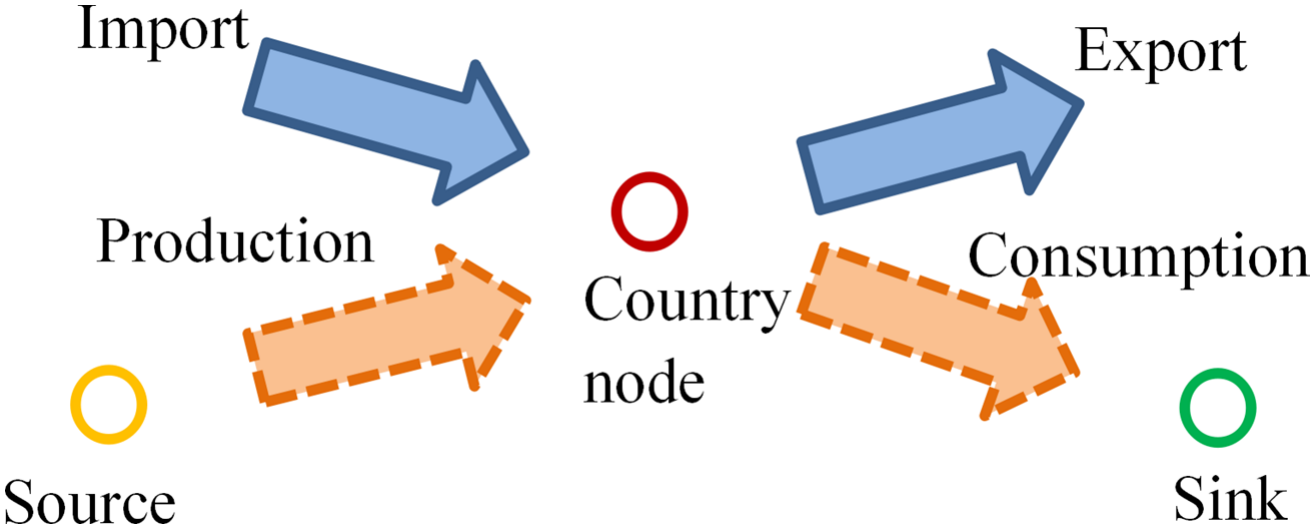
Illustration of a balanced country node from the viewpoint of commodity flow.

Besides, for each country *j* (as shown in Fig 2), according to the constraint of balanced inflow and outflow, the following equation should be satisfied:

$$\begin{array}{c} \sum_{i=1}^{N}e_{ij}+e_{0j}=\sum_{k=1}^{N}e_{jk}+e_{j,N+1}, \end{array}$$ (7)

where *e*_*ij*_ and *e*_*jk*_ are the value of the commodity inflow from *i* and the commodity outflow to *k* respectively, and *e*_0*j*_ and *e*_*j*,*N*+1_ are the value of the production and the consumption of country *j* respectively. If *e*_0*j*_ and *e*_*j*,*N*+1_ are not available, for simplicity, *e*_0*j*_ and *e*_*j*,*N*+1_ can be estimated as below:

$$\begin{array}{c} \left\{ \begin{array}{cc} e_{0j}=0;\;e_{j,N+1}=\sum_{i=1}^{N}e_{ij}-\sum_{k=1}^{N}e_{jk} & \;\text{if}\sum_{i=1}^{N}e_{ij}\geq \sum_{k=1}^{N}e_{jk},\\ e_{j,N+1}=0;\;e_{0j}=\sum_{k=1}^{N}e_{jk}-\sum_{i=1}^{N}e_{ij} & \;\text{if}\sum_{i=1}^{N}e_{ij}<\sum_{k=1}^{N}e_{jk}. \end{array}\right. \end{array}$$ (8)

For instance, for the commodity flow network of trading live bovine animals, node “China” has the commodity inflows from “Australia” and “Canada”, and the sum of values is 2,225 thousands of USD. Meanwhile, “China” has the commodity outflows to the countries (or areas), such as “Korea D P Rp”, and the sum of values is 19,724 thousands of USD. Since the production and the consumption of the commodity for “China” is unavailable, they can be estimated using Eq (8). Therefore, the values of the inflow from “source” to “China” and the outflow from “China” to “sink” are estimated as 17,499 thousands of USD and 0 respectively.

#### Viewpoint of Money Flow for Trading

Besides the viewpoint of trading commodity flow, the OFNs of international trade also can be established from the viewpoint of money flow. Because the trading of commodity is always accompanied by money flow, the direction of money flow is just the opposite of that of commodity flow, which is from the importer to the exporter; the volume flux of money flow is the same as that of commodity flow.

In a money flow network, node “source” and “sink” are the start and the end of all money flows respectively. Therefore, the edge from “source” to the country node *j* can represent country *j*’s trade deficit driven by the consumption of the commodity, and the edge from the country node *j* to “sink” represents the surplus of the exports over the imports.

**Fig 3:**
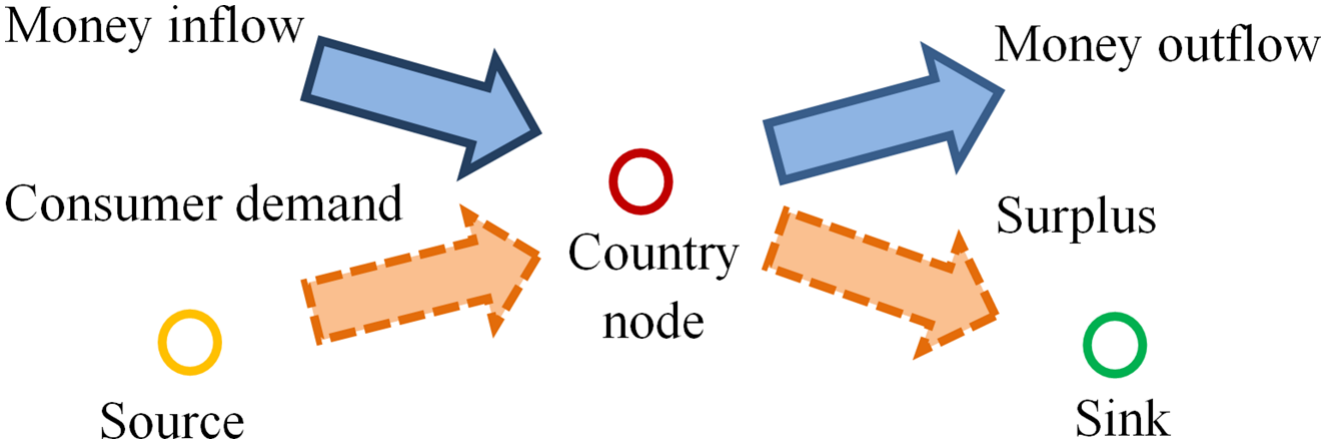
Illustration of a balanced country node from the viewpoint of money flow.

For each balanced country node *j* (as shown in Fig 3), the constraint of Eq (7) still holds, where *e*_*ij*_ and *e*_*jk*_ are the money inflow from country *i* to country *j* and the money outflow from country *j* to country *k* respectively. *e*_0*j*_ and *e*_*j*,*N*+1_ are the deficit and the surplus respectively, which also can be calculated using Eq (8).

For example, for the money flow network of trading live bovine animals, node “China” has the money outflows to “Australia” and “Canada”, whose sum of values is 2,225 thousands of USD; “China” also has the money inflows from the countries (or areas) such as “Korea D P Rp”, whose sum of values is 19,724 thousands of USD. It can be confirmed that the direction of money flow is the opposite of that of commodity flow. The value of the money inflow from “source” to “China” and that of the outflow from “China” to “sink” are 0 and 17,499 thousands of USD respectively. It means that “China” has the surplus of 17,499 thousands of USD for the trading of trading live bovine animals.

## Results

### Trading trophic levels

#### Countries’ trophic levels for a certain product

“Trophic level” is a term borrowed from ecology, meaning the position that a species occupies in a food chain. In international trade, we use this variable to quantitatively indicate a country’s role and position in the global supply chains (or the value-added chains), and its value is the country’s distance from “source”. Since there may be multiple paths from “source” to the country node, adopting the distance of the shortest path from “source” may underestimate the “trophic level” of the country. We consider that using the mean first-passage flow distance (MFPFD) from “source” is more reasonable, because this distance reflects the trade topology and flow dynamics in the OFNs [14].

**Fig 4:**
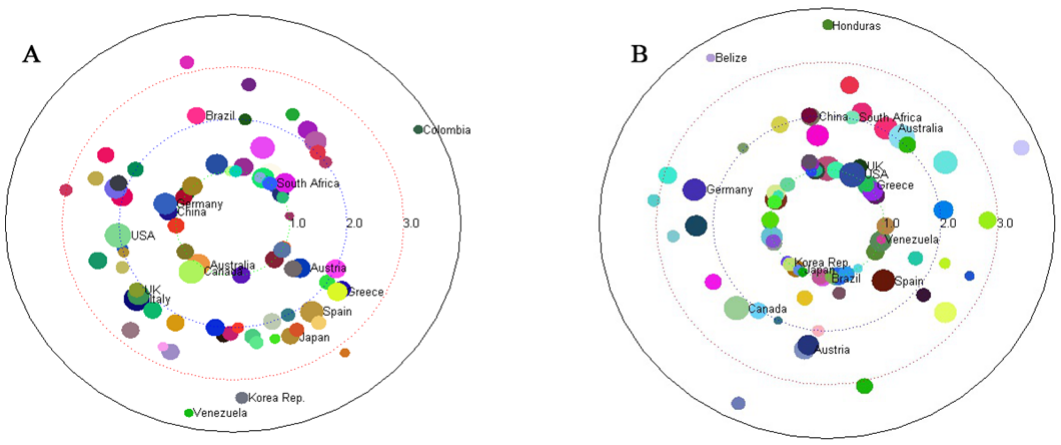
Trading trophic levels of countries for the commodity of live bovine animals from different viewpoints. (A) The viewpoint of commodity flow. (B) The viewpoint of money flow. Country nodes are plotted in a circle, whose distances to the center of the circle are proportional to their trophic levels, and whose angles and colors are selected randomly. The nodes sizes are in proportion to the natural logarithm of the nodes total outflow.

We use the OFN of live bovine animals (a commodity) as an example. Based on the MFPFD discussed in the section of materials and methods, we use the MFPFD from “source” to each country node as its trading trophic level. The trophic levels of countries from the viewpoints of commodity flow and money flow are calculated and then illustrated in Fig 4, respectively. In the figure, country nodes are plotted in a circle, whose distances to the center of the circle are proportional to their trophic levels, and whose angles and colors are selected randomly. The nodes’ sizes are in proportion to the natural logarithm of the nodes’ total outflow.

**Fig 5:**
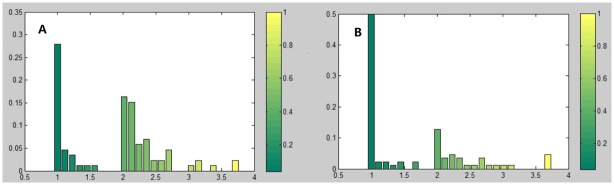
The distribution of trading trophic levels of countries for the commodity of live bovine animals. (A) The viewpoint of commodity flow. (B) The viewpoint of money flow.

From the viewpoint of commodity flow, the source of commodity flow is the production of this commodity and the sink means the consumption. So for a certain commodity, the trophic level of a country node represents the position that the country occupied in the global supply chains (i.e., the commodity flows). The smaller the trophic level, the role of the country is more inclined to be the producer (i.e., the exporter) of this commodity. On the contrary, the bigger the trophic level, the greater the distance from the source to the country node and the country is more inclined to be the consumer (that is the importer) of this commodity. From Fig 4A, we find that some countries (such as Germany, China, Australia, Canada and South Africa), whose trading trophic levels are slightly greater than or equal to 1, are inclined to be the exporters of live bovine animals. Some other nodes, such as Korea Rep., Venezuela and Colombia, which are far away from the centre of the circle, are the importers of live bovine animals. The corresponding distribution of trophic levels of countries is given in Fig 5A. Obviously, there are three major groups of countries, i.e., the countries whose trophic levels are between 1 and 1.6, between 2 and 2.8, and larger than 3. The universality of this pattern has been confirmed for a large variety of commodities.

From the viewpoint of money flow, the source is consumer demand for the commodity which leads to trade deficit, and the sink means the surplus. Because money flow can be obtained through interchanging “source” and “sink” and reversing the direction of edges of commodity flow network, it is intriguing to find some regularities and rules between the money flow network (say *MFN*) and the commodity flow network (say *CFN*) for a certain commodity. These empirical regularities include the followings.

1. For the sake of simplicity, the MFPFD from the source to the sink (i.e., *l*_*Source*,*Sink*_) is named *flow network length*. Then, for a certain commodity, its *MFN* and *CFN* have the same flow network length. For instance, for the commodity of live bovine animals, their flow network lengths are both 3.3674.
2. For a country node *i*, its trophic level on the *MFN* (i.e., $l_{Source,i}^{MFN}$) equals the MFPFD from *i* to the sink on the *CFN* (i.e., $l_{i,sink}^{CFN}$), and vice versa. For instance, for node “China”, we have $l_{Source,China}^{MFN}=l_{China,sink}^{CFN}=2$ and $l_{Source,China}^{CFN}=l_{China,sink}^{MFN}=1.16$.

The proofs of the above regularities can be found at S1 Text.

#### Comparing countries’ trading trophic levels for different products

**Fig 6:**
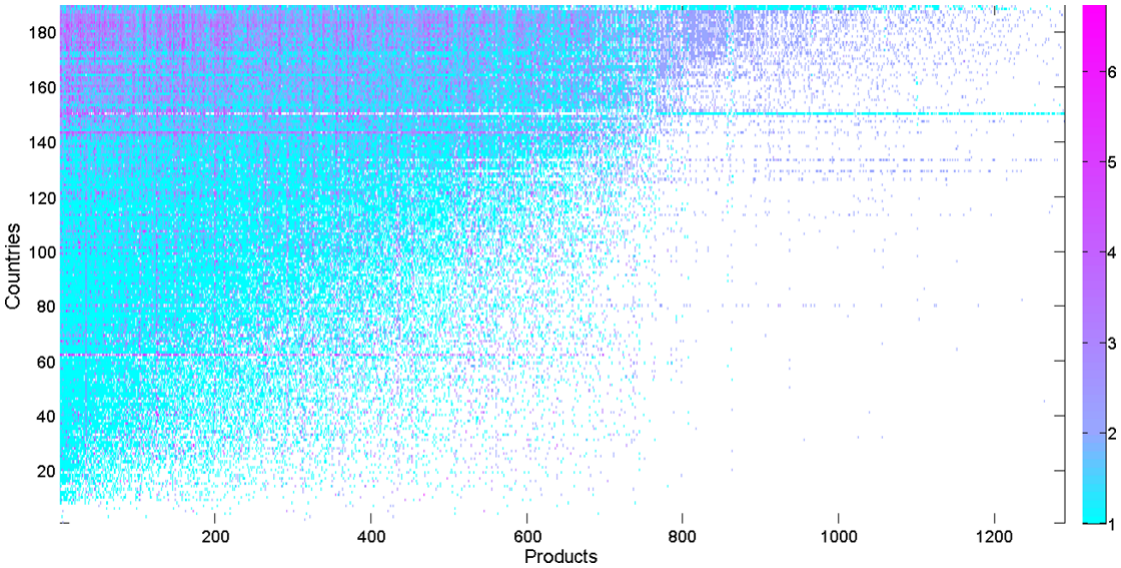
Countries-products matrix reporting countries’ trophic levels in different OFNs with different products. Rows (countries) and columns (products) are arranged in descending order of *n*, and then $\bar{l_{Source,i}^{\alpha }}$, where *n* and $\bar{l_{Source,i}^{\alpha }}$ are the number of colored points and the mean of trading trophic levels respectively.

We compare countries’ trophic levels in different OFNs with different products. We portray them using the countries-products matrix as shown in Fig 6. In the figure, each point represents a trophic level with the corresponding country and product, and its value is depicted by the color, where white indicates the value does not exist, cyan means the value is between 1 and 2 and indicates the country tends to be an importer, and magenta represents a relatively high value indicating an exporter. Two variables, i.e., the number of cyan and magenta points (say *n*) and the mean of trading trophic levels (say $\bar{l_{Source,i}^{\alpha }}$), are extracted to characterize each row (or column). Rows and columns of the matrix displayed in the figure are sorted by *n* in descending order, then by $\bar{l_{Source,i}^{\alpha }}$.

In Fig 6, we obtain an approximately right angle trapezoidal shape for cyan and magenta points. We can find that magenta points are concentrated in the upper portion of the trapezoidal shape, while the lower part is mainly cyan dots. It can be interpreted that the exporters of a wide spectrum of products are active countries in the international trade market, which are located in the upper part of the figure and can be regarded as competitive and success countries according to [25]. In contrast, bottom rows are those not active in the international trading, which can only export a limited variety of products and tend to be underdeveloped countries [25]. Economies, which export few types of goods but import a large variety of products, also can occur in the upper part of the figure. Examples include Saudi Arabia and United Arab Emirates. Besides, we also find some abnormal countries, which appear as cyan horizontal lines in the top-right of the figure. It can be interpreted that these countries import many kinds of goods, which the vast majority of countries do not need to import. It may indicate that these economies are at high risk. A representative example is Yugoslavia in the year 2000.

### Trading niches

#### Countries’ trading niches for a certain product

Since trophic level only uses the distance from the source to depict the role and position of a country in the trading, a more comprehensive approach is to consider both the distances from the source and to the sink. Therefore, we use the term “trading niche” to more precisely capture a country’s role and position in the global supply chains (or value-added chains). It comprises two dimensions, the MFPFD from the source (i.e., *l*_*Source*,*i*_) and that to the sink (i.e., *l*_*i*,*Sink*_), where the first one is exactly trading trophic level.

**Fig 7:**
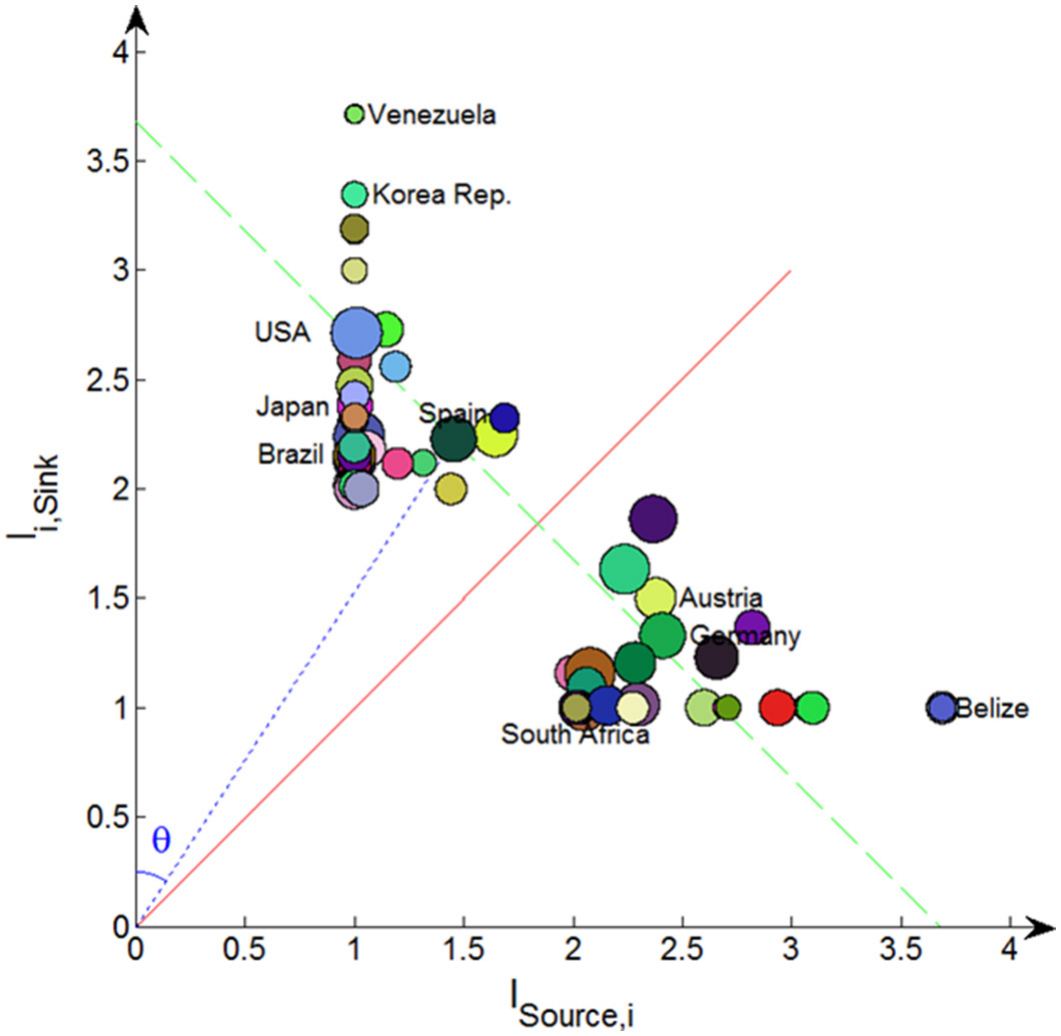
Trading niches of countries for the commodity of live bovine animals from the viewpoint of money flow. The red diagonal line separates importers and exporters. *θ* is the angle between vertical axis and the line connecting the origin and country node.

Fig 7 illustrates trading niches for the commodity of live bovine animals from the viewpoint of money flow, where the x-coordinate is *l*_*Source*,*i*_ and the y-coordinate is *l*_*i*,*Sink*_ of a country. The role of a country as an importer (or exporter) in the trading can be indicated by the following variable, which is named as *trade role indicator*.

$$\begin{array}{c} Tan\left(\theta \right)=l_{Source,i}/l_{i,Sink} \end{array}$$ (9)

where *θ* is the angle between vertical axis and the line connecting the origin and the country node. For a given node, if its trade role indicator (*Tan*(*θ*)) is bigger than 1 (i.e., *l*_*Source*,*i*_ > *l*_*i*,*Sink*_), it is an exporter; the larger the value, the more it tends to be a pure exporter. If the trade role indicator (*Tan*(*θ*)) is smaller than 1, it can be regarded as an importer; the smaller the value, the country is more inclined to be a pure importer.

From Fig 7, we find that these countries are naturally divided into two groups: countries in the top left and those in the bottom right of the red diagonal line, which are importers and exporters of the commodity respectively. Although both South Africa and Belize have the same *l*_*i*,*Sink*_, a larger *θ* makes the latter more likely a pure exporter. Compared with USA, Venezuela is a more pure importer, because it has a smaller *θ*.

The value of *l*_*Source*,*i*_ + *l*_*i*,*Sink*_ is also meaningful, which can be explained as below. Suppose a large enough number (say *λ*) of particles flow along directed edges randomly. *l*_*Source*,*i*_ + *l*_*i*,*Sink*_ is the expected number of steps for the particles starting from “source”, passing through *i* and finally reaching “sink” for the first time. Therefore, we call it *flow network length of passing through i*.

To visualize the value of *l*_*Source*,*i*_ + *l*_*i*,*Sink*_ in the figure, we establish the following linear equation.

$$\begin{array}{c} y=-x+(l_{Source,i}+l_{i,Sink}) \end{array}$$ (10)

For each node *i*, a line of the above equation can be drawn, and *l*_*Source*,*i*_ + *l*_*i*,*Sink*_ can be represented as the y-intercept of the line. The line divides countries into three groups. Countries in the line have the same value of *l*_*Source*,*i*_ + *l*_*i*,*Sink*_; countries in the bottom left of the line have a smaller *l*_*Source*,*i*_ + *l*_*i*,*Sink*_; other countries in the top right of the line have a larger value. In Fig 7, the line for Spain is illustrated with a green dashed line. We find USA has the same value of *l*_*Source*,*i*_ + *l*_*i*,*Sink*_ with Spain; the flow network length of passing through Venezuela is larger, and that of South Africa is smaller.

#### Synthetic analysis of countries’ trading niches for different products

We calculate countries’ trading niches in different OFNs with different products, and then generate the corresponding *Tan*(*θ*) and *l*_*Source*,*i*_ + *l*_*i*,*Sink*_, which are stored in two countries-products matrices.

Then, we define a country’s *synthetic trade role indicator* (i.e., *Tan*(*ξ*)) for a category of commodities as below.

$$\begin{array}{c} Tan\left(\xi \right)=\sqrt[{n}]{Tan\left(\theta_{1}\right)\times Tan\left(\theta_{2}\right)\times \cdots \times Tan\left(\theta_{n}\right)} \end{array}$$ (11)

where *Tan*(*θ*_1_), *Tan*(*θ*_2_), ⋯*Tan*(*θ*_*n*_) are trade role indicators for commodities in this category. According to Eq (11) and the discussion of *Tan*(*θ*) in the previous subsection, it is easy to derive that the value of 1 can be considered as the watershed for *Tan*(*ξ*): the more the value of *Tan*(*ξ*) is greater than 1, the more the country tends to be a pure exporter; in contrast, the more the value is less than 1, the more the country is inclined to be a pure importer. Thus, in Table 2, we list some representative countries with their *Tan*(*ξ*) for different categories of commodities, where the classification is given by SITC4 [23]. For Argentina, it is a typical agricultural country, which exports primary products (categories 0, 1, 2, 3, 4) and imports manufactured products (categories 5, 6, 7 and 8). In contrast, Japan is a typical industrial country, which imports primary products and exports manufactured products. Gambia is one of least developed countries, whose *Tan*(*ξ*) are almost all far less than 1. It means that Gambia imports all categories of products. In contrast, Netherlands is a representative export-oriented developed country, which is able to export almost all categories of products. USA is a developed country with a balanced role between importer and exporter, whose *Tan*(*ξ*) are all around 1.

**Table 2:** Representative countries with their synthetic trade role indicators for different categories of commodities.

Code	Classification	Argentina	Japan	Gambia	Netherlands	USA
0	Food and live animals	1.39	0.49	0.57	1.21	1.00
1	Beverages and tobacco	1.31	0.47	0.35	1.21	0.91
2	Crude materials, inedible, except fuels	1.12	0.57	0.90	1.08	1.05
3	Mineral fuels, lubricants and related materials	0.98	0.73	0.34	1.09	0.94
4	Animal and vegetable oils, fats and waxes	1.16	0.55	0.35	1.25	0.93
5	Chemicals and related products, n.e.s.	0.53	1.15	0.26	1.47	1.26
6	Manufactured goods classified chiefly by material	0.55	1.16	0.38	1.06	0.81
7	Machinery and transport equipment	0.42	1.67	0.33	1.17	1.00
8	Miscellaneous manufactured articles	0.43	0.99	0.39	0.95	0.85

### Node centrality

#### Countries’ node centralities for a certain product

Given a certain product, we calculate country centrality based on flow distances in the flow network. The centrality of a country node can be computed as the average of distances from the node to all the other countries as given in [14]. However, if one of those distances is infinite, the node centrality will become infinite. Thus, the above computation method is infeasible for a sparse distance matrix in which most of the elements are infinite. Therefore, we propose a new definition of node centrality, called harmonic centrality, as

$$\begin{array}{c} f_{i}=\frac{N-1}{\frac{1}{f_{i1}}+\frac{1}{f_{i2}}+\cdots +\frac{1}{f_{i,i-1}}+\frac{1}{f_{i,i+1}}+\cdots +\frac{1}{f_{i,N-1}}+\frac{1}{f_{i,N}}}, \end{array}$$ (12)

where *f*_*i*,*j*_ (*j* = 1, ⋯, *N* and *j* ≠ *i*) is the SMFDs between nodes *i* and *j*, and *N* is the number of common nodes in the flow network. Two special nodes, i.e., source node 0 and sink node *N* + 1, are excluded, because here we are concerned about the distances of the node to the other common nodes, and the distances to the source and the sink have been depicted in trading trophic levels and niches.

Thus, the harmonic node centrality metric can avoid the infinite distance problem and well measure centralities of nodes. A smaller *f*_*i*_ implies a more central position that the node occupied in the flow network, because it has a smaller average distance to the other nodes. In Table 3, we list the top 5 and the bottom 5 countries with their *f*_*i*_ in the decreasing order of *f*_*i*_ in the money flow network of trading live bovine animals. The top 5 countries are all important hub nodes from the viewpoint of topology.

**Table 3:** List of countries sorted by *f*_*i*_ for the money flow network of trading live bovine animals.

Top 5 countries			Bottom 5 countries		
Rank	Country	*f*_*i*_	Rank	Country	*f*_*i*_
1	Germany	2.53	82	Ecuador	86.00
2	Netherlands	2.74	83	Mozambique	86.00
3	Hungary	3.05	84	Bahrain	86.00
4	Australia	3.10	85	Qatar	86.00
5	Italy	3.15	86	Singapore	86.00

#### Comparing countries’ centralities for different products

We compute and compare node centralities of countries in different OFNs with different products. Firstly, we establish a countries-products matrix *F* = {*f*_*i*,*j*_}_*M*×*N*_, where *f*_*i*,*j*_ is the corresponding harmonic centrality of country *i* (*i* ∈ {0, 1, ⋯, *M* − 1}) for the commodity *j* (*j* ∈ {0, 1, ⋯, *N* − 1}), *M* is the total number of countries and *N* is the total number of types of commodities. And then, we transform it into a ranking matrix *R* = {*r*_*i*,*j*_}_*M*×*N*_, where *r*_*i*,*j*_ is the ranking of country *i*’s centrality among the elements in the *j*-th column, which are countries’ centralities for commodity *j*.

**Fig 8:**
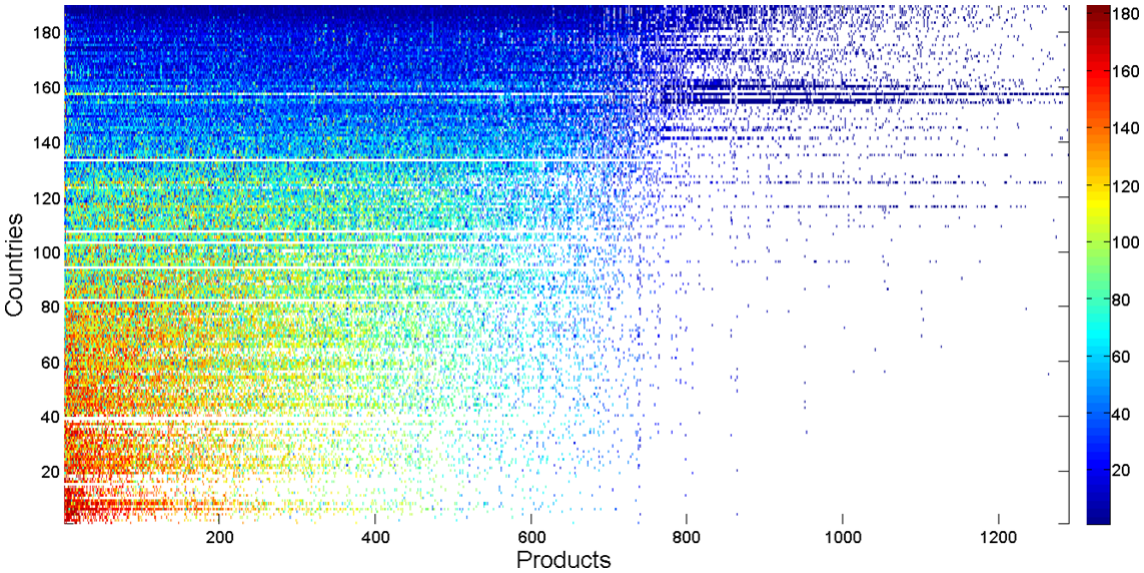
Countries-products matrix reporting countries’ harmonic centrality rankings in columns. Countries are sorted in ascending order by the average ranking, and products are arranged in descending order of the number of trading countries.

For the purpose of visualization, we rearrange the rows and columns of *R* and obtain a sorted matrix $R^{\prime }={\left\{r_{i,j}^{\prime }\right\}}_{M\times N}$, where rows (countries) are sorted in ascending order by the average of countries’ rankings (i.e., $\bar{\sum_{j}r_{i,j}}$), and columns (products) are arranged in descending order of the number of trading countries. The sorted ranking matrix is illustrated in Fig 8, where each point represents the corresponding ranking $r_{i,j}^{\prime }$ and its value is depicted by the color. As shown in the colormap of Fig 8, the color gradually changes from blue to cyan to yellow to red, as the ranking goes from high to low; white indicates the value does not exist. From the figure, we get an approximately right angle trapezoidal shape for colored dots, which is quite similar to the shape in Fig 6. It can be interpreted that active countries which trade a wide spectrum of products are likely to have high average harmonic centrality; on the contrary, those having few types of trading products tend to have low average rankings. Most interestingly, in the figure there are several horizontal color bands, which are blue, cyan, yellow and red successively from top to bottom. This phenomenon can be considered as *centrality stratification*. It indicates that competitive countries tend to be in the center position in the trading of a large variety of products, while underdeveloped countries likely rank low in their limited varieties of trading products. In Table 4, we list the top 8 and bottom 8 economies with their corresponding average harmonic centrality rankings (say ${\bar{f}}_{i}$) and numbers of types of trading products (say *n*). It implies that ${\bar{f}}_{i}$ may be a good alternative indicator for countries’ competitiveness from the perspective of node centrality, and countries are encouraged to promote their harmonic centrality rankings in the trading of various products for competitiveness enhancement.

**Table 4:** List of economies sorted by the average harmonic centrality ranking. Note: *n* is the number of types of trading products and ${\bar{f}}_{i}$ is the country’s average harmonic centrality ranking.

Top 8 economies				Bottom 8 economies			
Rank	Economy	*n*	${\bar{f}}_{i}$	Rank	Economy	*n*	${\bar{f}}_{i}$
1	Germany	928	4.95	183	Guinea-Bissau	268	126.72
2	USA	992	5.47	184	Chad	129	126.94
3	France, Monac	946	6.86	185	Greenland	299	127.99
4	UK	938	7.06	186	Burundi	108	128.60
5	Italy	917	8.04	187	Samoa	123	133.15
6	China	914	11.43	188	Occ. Pal. Terr	61	134.02
7	Spain	912	12.13	189	St.Pierre Mq	68	134.88
8	Netherlands	927	13.01	190	CACM NES	21	137.86

## Conclusions and Discussions

With the acceleration of the process of globalization, countries are increasingly involved in cross-border trade activities. The value and tonnage of international trade flows are accelerating growth, and the impact of international trade flows in the global economy is also growing [1, 26]. Therefore, it is important and necessary to explore the global trade flow system in depth.

In this paper, we use the perspective of open flow networks to model international trade flow system, and provide the approaches of flow distances for investigating them. The novel model with its approaches offers us an effective tool for quantitatively exploring the roles and positions of countries in the global supply chains (or value-added chains). We introduce the formal description of OFNs of international trade, and flow distances on them (e.g., the mean first-passage flow distance and the mean total flow distance), where a new flow distance called symmetric minimum flow distance is proposed for the application need of symmetric distance metrics. Then, we present two coupled viewpoints for establishing OFNs of international trade, i.e., the viewpoint of commodity flow and that of money flow. Thus, based on the perspective of flow network and the approaches of flow distances, insights are gained into international trade flows.

Firstly, countries’ trading “trophic levels” and “niches” are used to depict the roles and positions that countries occupied in the international supply chains (or the value-added chains). From the distribution of trading “trophic levels”, we find that countries can be divided into three groups: countries whose trophic levels are slightly bigger than or equal to 1, between 2 and 3, and larger than 3. Besides, since the viewpoints of commodity flow and money flow are coupled, some regularities can be found. For example, money flow network and commodity flow network for the same commodity have the same flow network length, which is the mean first-passage flow distance from “source” to “sink”.

Secondly, by comparing countries’ trophic levels in different OFNs with different products, we find that exporters of a wide spectrum of products are active and competitive countries in the international trade, while countries export a limited types of commodities are inactive and tend to be underdeveloped countries. Besides, we find some countries import many kinds of goods, which the vast majority of countries do not need to import. This phenomenon may indicate that these economies are at high economic risk.

Thirdly, based on trading niches, a country’s trade role indicator for a certain commodity and its synthetic version for a category of commodities are proposed. The value of 1 is the watershed for trade role indicator: the more the value is greater than 1, the more the country tends to be a pure exporter; in contrast, the more the value is less than 1, the more the country is inclined to be a pure importer. Some representative countries with their indicators are exhibited. For example, USA is a country with a balanced role between importer and exporter, whose values of synthetic trade role indicators for various categories of commodities are all around 1.

Fourthly, we propose a new node centrality, called harmonic centrality, for solving the problem of infinite distance. A smaller harmonic centrality indicates a more central position the node occupied. Thus, each country’s centrality in a flow network system can be quantitatively calculated and ranked. Then, we compare harmonic centralities of countries in the OFNs with different products. It is interesting to find the phenomenon of centrality stratification. It means that competitive countries tend to be in the center position in the trading of a large variety of products, while underdeveloped countries likely rank low in their limited varieties of trading products.

Our findings demonstrate the effectiveness of the proposed model of OFNs and the approaches of flow distances for investigating countries’ roles and positions in the trade flow network system. Based on this novel model, more flow network approaches (such as cascading failure analysis [27]) can be introduced to (or developed for) the study of international trade, and more insights can be obtained into the global trade flows.

## Supporting Information

S1 Text Proofs. Proofs of the empirical regularities between the money flow network and the commodity flow network for a certain commodity.
(DOCX)

## References

[1] Manyika J, Bughin J, Lund S, Nottebohm O, Poulter D, Jauch S, et al. Global flows in a digital age: How trade, finance, people, and data connect the world economy. McKinsey Global Institute; 2014.

[2] Ricardo D. On the principles of political economy and taxation John Murray, London; 1817.

[3] Frankel JA, Romer D. Does trade cause growth? American economic review. 1999; 89(3): 379–399. 10.1257/aer.89.3.379

[4] Milani F, Park SH. The effects of globalization on macroeconomic dynamics in a trade-dependent economy: The case of Korea. Economic Modelling. 2015; 48: 292–305. 10.1016/j.econmod.2014.10.042

[5] Hausmann R, Hidalgo CA. The network structure of economic output. Journal of Economic Growth. 2011; 16(4): 309–342. 10.1007/s10887-011-9071-4

[6] Fan Y, Ren S, Cai H, Cui X. The state’s role and position in international trade: A complex network perspective. Economic Modelling. 2014; 39: 71–81. 10.1016/j.econmod.2014.02.027

[7] Tinbergen J. Shaping the world economy; suggestions for an international economic policy. Twentieth Century Fund; 1962.

[8] Baltagi BH, Egger P, Pfaffermayr M. A generalized design for bilateral trade flow models. Economics Letters. 2003; 80(3): 391–397. 10.1016/S0165-1765(03)00115-0

[9] Ghironi F, Melitz MJ. Trade flow dynamics with heterogeneous firms. The American economic review. 2007; 97: 356–361. 10.1257/aer.97.2.356

[10] Helpman E, Melitz M, Rubinstein Y. Estimating trade flows: Trading partners and trading volumes. The Quarterly Journal of Economics. 2008; 132(2): 441–487. 10.1162/qjec.2008.123.2.441

[11] Serrano MÁ, Boguñá M, Vespignani A. Patterns of dominant flows in the world trade web. Journal of Economic Interaction and Coordination. 2007; 2(2): 111–124. 10.1007/s11403-007-0026-y

[12] Serrano MÁ, Boguñá M. Topology of the world trade web. Physical Review E. 2003; 68(1): 015101 10.1103/PhysRevE.68.015101 12935184

[13] Fagiolo G, Reyes J, Schiavo S. On the topological properties of the world trade web: A weighted network analysis. Physica A: Statistical Mechanics and its Applications. 2008; 387(15): 3868–3873. 10.1016/j.physa.2008.01.050

[14] Guo L, Lou X, Shi P, Wang J, Huang X, Zhang J. Flow Distances on Open Flow Networks; 2015. Preprint. Available: arXiv:1501.06058v1. Accessed 17 March 2015.

[15] Gallos LK, Song C, Havlin S, Makse HA. Scaling theory of transport in complex biological networks. Proceedings of the National Academy of Sciences. 2007; 104(19): 7746–7751. 10.1073/pnas.0700250104 PMC1876518 17470793

[16] Zhang J, Guo L. Scaling behaviors of weighted food webs as energy transportation networks. Journal of theoretical biology. 2010; 264(3): 760–770. 10.1016/j.jtbi.2010.03.024 20303987

[17] Zhang J, Wu L. Allometry and dissipation of ecological flow networks. PloS one. 2013; 8(9): e72525 10.1371/journal.pone.0072525 24019871 PMC3760856

[18] Khan SI, Maini P. Modeling heterogeneous traffic flow. Transportation Research Record. 2007; 1678(1): 234–241.

[19] Ser-Giacomi E, Rossi V, López C, Hernández-García E. Flow networks: A characterization of geophysical fluid transport; 2014. Preprint. Available: arXiv:1409.4171v2. Accessed 15 January 2015;. 10.1063/1.4908231 25833442

[20] Ahuja RK, Magnanti TL, Orlin JB. Network flows: theory, algorithms, and applications. Prentice Hall, Upper Saddle River, New Jersey; 1993.

[21] Rosvall M, Bergstrom CT. Maps of random walks on complex networks reveal community structure. Proceedings of the National Academy of Sciences. 2008; 105(4): 1118–1123. 10.1073/pnas.0706851105 PMC2234100 18216267

[22] Vézina AF, Platt T. Food web dynamics in the ocean. I. Best-estimates of flow networks using inverse methods. Marine Ecology—Progress Series. 1988; 42(3): 269–287.

[23] United Nations Statistics Division. Detailed structure and explanatory notes: Standard International Trade Classification, Rev.4; 2014. Available from: http://unstats.un.org/unsd/cr/registry/regcst.asp?Cl = 28

[24] Feenstra RC, Lipsey RE, Deng H, Ma AC, Mo H. World trade flows: 1962–2000. National Bureau of Economic Research; 2005. Available from: http://www.nber.org/papers/w11040

[25] Tacchella A, Cristelli M, Caldarelli G, Gabrielli A, Pietronero L. A new metrics for countries’ fitness and products’ complexity. Scientific reports. 2012; 2: 723 10.1038/srep00723 23056915 PMC3467565

[26] Dreher A. Does globalization affect growth? Evidence from a new index of globalization. Applied Economics. 2006; 38(10): 1091–1110. 10.1080/00036840500392078

[27] Allesina S, Bodini A. Who dominates whom in the ecosystem? Energy flow bottlenecks and cascading extinctions. Journal of Theoretical Biology. 2004; 230(3): 351–358. 10.1016/j.jtbi.2004.05.009 15302545

