# Supplementary material for: International Trade Modelling Using Open Flow Networks: A Flow-Distance Based Analysis
Source: PLoS One. 2015 Nov 16;10(11):e0142936. doi: 10.1371/journal.pone.0142936 (PMC4646344; doi:10.1371/journal.pone.0142936)
Supplement: S1 Text — Proofs of the empirical regularities between the money flow network and the commodity flow network for a certain commodity. (DOCX) [file pone.0142936.s001.docx]

**Proofs.**

**Proofs of empirical regularities between the money flow network and the commodity flow network for a certain commodity**

Given a commodity, we can obtain a commodity flow network (say *CFN*) and a money flow network (say *MFN*). The structures of *CFN* and *MFN* are coupled, and have the following properties.

Property 1. The topologies of *CFN* and *MFN* are the same.

Property 2. In *CFN* and *MFN*, the volume fluxes of the corresponding edges are the same, but the directions are completely reversed.

Property 3. The source and the sink in these two networks are interchanged.

These properties have been given and proved in the main text (Page 5). In the following, we further prove two propositions about *CFN* and *MFN*, which are presented in the main text (Page 8).

**Proposition 1.** For a certain commodity, its money flow network (*MFN*) and commodity flow network (*CFN*) have the same flow network length.

Proof. Given an open flow network (*OFN*), suppose a large enough number (say $\lambda$) of particles start from the source and end at the sink, which flow along directed edges randomly. For each edge, we can obtain its corresponding volume flux. Let us mark particles' flow paths from the source to the sink as $path_{i}$, $i=1,\text{ }2,\cdots,\text{ }\lambda$. Then, according to the definition of the mean first-passage flow distance (MFPFD), we have

$l_{Source,\text{ }Sink}^{OFN}=\sum_{i} step(path_{i})/\lambda$, (1)

where $l_{Source,\text{ }Sink}^{OFN}$ is the flow network length of *OFN*, and $step(path_{i})$ is the number of steps for *path_i_*.

Suppose we reverse the directions of all edges and interchange the source and the sink. Besides, we reverse the original path of each particle *i* to keep the volume flux of each edge unchanged. Thus, we obtain a reversal of *OFN* (say $OFN'$)[[1]](#footnote-0). Therefore, the flow network length of $OFN'$ can be computed as below.

. (2)

where $path'_{i}$ is the reversal of $path_{i}$.

Because the number of steps for *path_i_* is equal to that of $path'_{i}$, that is to say, $step(path_{i})=step(path'_{i})$, based on Equations 1 and 2, we have

$l_{Source,\text{ }Sink}^{OFN}=l_{Source,\text{ }Sink}^{OFN'}$. (3)

For a certain commodity, its *MFN* is exactly the reversal of *CFN*, where *MFN* and *CFN* have Properties 1, 2 and 3 (see Page 5 of the main text). Therefore, according to Equation 3, *MFN* and *CFN* have the same flow network length.

**Proposition 2.** For a country node *i*, its trophic level on the *MFN* (i.e., $l_{Source,\text{ }i}^{MFN}$) equals the MFPFD from *i* to the sink on the *CFN* (i.e., $l_{i,\text{ }Sink}^{CFN}$), and vice versa.

Proof. Given an open flow network (*OFN*), suppose a large enough number (say $\lambda$) of particles flow from the source to node *i* along directed edges randomly. Mark the flow path for particle *j* as $path_{j}$,$j=1,2,\cdots,\lambda$. Then, according to the definition of the mean first-passage flow distance (MFPFD), we have

$l_{Source,\text{ }i}^{OFN}=\sum_{j} step(path_{j})/\lambda$, (4)

where $step(path_{j})$ is the number of steps for *path_j_*.

Suppose $OFN'$ is exactly the reversal of *OFN*, where the source and the sink are interchanged and the directions of flows are reversed. Besides, the volume fluxes of edges need to be kept unchanged. Therefore, in $OFN'$, $\lambda$ particles flow from node *i* to the sink, and the flow path of each particle *j* (say $path'_{j}$) is just the reversal of its original path. Thus, we have

. (5)

Because $step(path_{j})=step(path'_{j})$, according to Equations 4 and 5, we have

$l_{Source,\text{ }i}^{OFN}$ =$l_{i,\text{ }Sink}^{OFN'}$ (6)

For a certain commodity, because its *MFN* is the reversal of *CFN* and vice versa, we have

$l_{Source,\text{ }i}^{MFN}$=$l_{i,\text{ }Sink}^{CFN}$, (7)

$l_{Source,\text{ }i}^{CFN}$=$l_{i,\text{ }Sink}^{MFN}$. (8)

Thus, Proposition 2 is proved.

1. For two open flow networks *OFN* and $OFN'$, if they have Properties 1, 2 and 3 (Lines 8 - 11 of the supplementary text), we call $OFN'$ is the reversal of *OFN* and vice versa. [↑](#footnote-ref-0)
